# ‘Internet+’ comprehensive nursing training course in the post-epidemic era—an exploration of the mixed teaching mode: a randomized trial

**DOI:** 10.3389/fmed.2023.1152732

**Published:** 2023-06-28

**Authors:** Qing-Ling Wang, Lan-Lan Liu, Cheng-Rui Liu, Qing-Shuang Zhu, Zhi-Ying Ren, Ting-Ting Gang, Chun-Yan Zhou, Qiu-Ping Li, Xi Chen, Bin-Ru Han

**Affiliations:** ^1^Department of Emergency, Xuanwu Hospital, Capital Medical University, Beijing, China; ^2^School of Economics and Management, Beijing University of Technology, Beijing, China; ^3^Education Division, Xuanwu Hospital, Capital Medical University, Beijing, China; ^4^Department of Nursing, Xuanwu Hospital, Capital Medical University, Beijing, China

**Keywords:** comprehensive nursing training, “internet+”, professional identity, self learning ability, exploration of mixed teaching mode

## Abstract

**Objective:**

To explore the effect of the application of the ‘Internet+’ nursing teaching mode on the comprehensive teaching ‘Fundamentals of Nursing’.

**Trial design:**

Parallel design and convenient sampling were used to select vocational nursing students from the Nursing College of Capital Medical University.

**Methods:**

Selected students were randomly divided into two groups. The control group consisted of 30 students in Grade 2020 higher vocational nursing education (traditional teaching mode). The observation group consisted of 30 students in Grade 2021 higher vocational nursing education (Internet+ mixed teaching mode). Training assessment results, automatic learning ability, professional identity, and satisfaction were compared between the two groups.

**Results:**

Compared with the control group, the students in the observation group scored higher in the following operation practices: venous blood sampling, intradermal injection, cardiopulmonary resuscitation (CPR), sputum aspiration, and putting on and taking off robes (84.01 ± 0.87 vs. 92.14 ± 1.23; 91.41 ± 0.82 vs. 96.86 ± 0.27; 87.56 ± 0.31 vs. 93.91 ± 2.79; 88.11 ± 0.51 vs. 93.75 ± 0.29; and 82.29 ± 0.29 vs. 90.96 ± 0.34, respectively, with *p* < 0.05 for all scores). The total scores for autonomous learning ability and subjective satisfaction were also higher in the observation group compared with the control group (82.98 ± 4.72 vs. 93.17 ± 5.01 and 96.67% vs. 90.00%, respectively, with *p* < 0.05 for all scores).

**Conclusion:**

In the post-epidemic era, the ‘Internet+ hybrid teaching mode’ was applied to comprehensive nursing teaching. This changed the traditional education mode, which focuses only on professional knowledge. The ‘Internet+’ teaching mode results showed that the professional, ideological, and political courses exhibited the same value guidance, which improved students’ independent learning ability, practical operation ability, professional identity, and satisfaction.

## 1. Introduction

The COVID-19 outbreak has confirmed that a shortage of nursing staff leads to difficulties in the prevention and control of an epidemic, as well as the recovery and treatment of patients. Therefore, training high-quality nurses and developing their professional identity is an urgent problem for nursing educators and society. A post-epidemic era refers to an era when planning and development occurs in preparation for a new epidemic in the future (1). Comprehensive nursing practice courses are highly practical and applicable. The essence of clinical practice teaching is that nurses use professional knowledge and skills to solve practical clinical problems, which directly affects the quality of nursing and patient safety. Clinical practice ability is a key indicator for testing the quality of nursing education and the effectiveness of clinical teaching (2, 3). There are many uncertainties in clinical nursing, which require students to possess professional ability and operational skills (4). Therefore, nursing training should focus on cultivating students’ clinical skills and emergency response abilities (5). Presently, in clinical practice, a standardized training mode is used, with competence improvement at its core. However, to a large extent, theoretical training alone is not sufficient for improving the adaptability and practical application ability of nursing staff in dealing with emergency clinical situations, where knowledge of both routine clinical procedures and emergency treatment procedures is required (6).

In recent years, the teaching methods of colleges and universities have also gradually deepened the reform. For example, a flipped classroom can be used with the help of the internet since its development is less limited by classroom time and location, achieving better results. Thus, the flipped classroom has been applied and promoted in recent years (7, 8). In addition, problem-based learning (PBL) teaching uses classic open cases as guides and asks questions. Students can carry out cooperative learning in groups to analyze problems encountered in these cases, promoting students to become explorers of learning, which can improve their learning initiative, enthusiasm, and application value (9). However, the teaching of comprehensive nursing training has encountered many new problems and challenges, such as how to connect online teaching with offline teaching, how to distribute the content, how to coordinate the teaching, and how online and offline teaching can complement each other. The online learning ability of students varies greatly; therefore, another challenge is how to maximize homogeneity (10, 11). Based on these problems and challenges, it is necessary to build a diversified, mixed teaching mode that conforms to the development of the times, integration of ideological and political education as well as professional knowledge, integration of traditional and modern education, and deep integration of information technology, education, and teaching. This type of teaching mode would effectively make up for the shortcomings of the single teaching method, thus meeting the needs of higher medical education in a post-epidemic era for training high-quality, comprehensive nursing talents (12). However, in the current epidemic situation, ‘Internet+’ education is facing unprecedented opportunities and challenges (13). The deep integration of modern information technology, education, and teaching can effectively change classroom organization, teaching processes, and student learning methods, while strengthening the student-centered position and promoting personalized learning, which can in turn significantly improve classroom teaching and student comprehension. Therefore, as a nursing educator, it is necessary to reflect on the basis of traditional teaching, as well as on how nursing teaching responds to the shift to the ‘Internet+’ education era. It is also necessary to provide new ideas to improve student quality and professional nursing identity. This study implements the standardized training model for nursing practice combined with ‘Internet+’, aiming to explore the effect of this teaching model on the training of nursing students.

## 2. Participants and methods

### 2.1. Experimental design

The parallel design research method was used to facilitate sampling and selection of students from the higher vocational nursing classes of 2020 and 2021 in Capital Medical University. Simple random sampling and the random number table method were used to divide the students into two groups. The control group consisted of nursing students from the 2020 higher vocational nursing class and the observation group of nursing students from the 2021 higher vocational nursing class. The inclusion criteria were students in the higher vocational nursing classes of 2020 and 2021 who had completed all Comprehensive Nursing Training courses. The exclusion criterion was students absent for over 10% of the class hours. The research purpose and method were explained to participants, and informed consent was obtained. The data collected were only used for the analysis conducted in this research, and formative evaluation did not consider students’ academic achievements. This study followed the CONSORT Guidelines for the design and implementation of randomized controlled trials (14) and was approved by the Ethics Approval Committee of the University (Approval No.: 2022JYY454).

The sample size of this study was determined by the practicalities of working in a tertiary hospital, where the intervention needed to be delivered over a period of one semester and at a time amenable to scheduling requirements and the clinical rotation of nursing students. Assuming a high correlation of 0.8 for baseline-*post hoc* measures (15, 16) and to detect a moderate effect size for the outcome variable (17), each group would need to consist of at least 30 students to ensure 90% power and a 5% significance level.

### 2.2. Teaching methods

**Figure 1:**
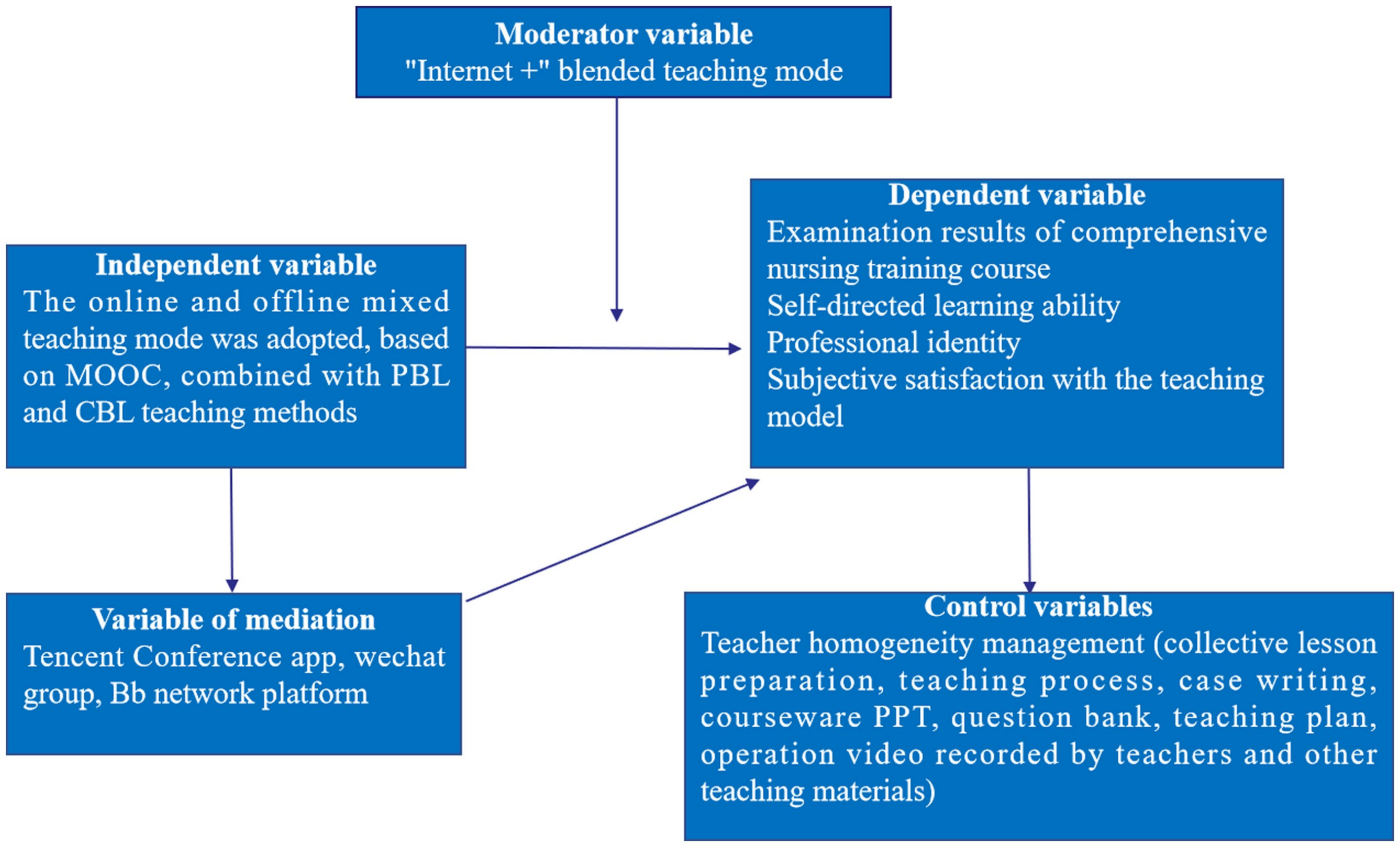
Theoretical framework.

The practical course adopted the inter-class practice method. The instructor taught each practical skill to small groups of 3–5 participants. The theoretical framework of this study is shown in Figure 1.

(1) The control group: For nursing students from the 2020 higher vocational nursing class, we carried out face-to-face teaching under the routine skill operation line; the teacher prepared the teaching plan according to the comprehensive nursing training syllabus, formulated the teaching schedule and class hours, and imparted theoretical and practical knowledge to students through PPT, on-site operation demonstration, blackboard writing, and other forms. The teacher demonstration teaching method included nursing student practise, anti-teaching, comments, and then re-practise.
(2) Observation group: For nursing students from the 2021 higher vocational nursing class, the online and offline hybrid teaching mode was adopted. The mixed teaching group used a method based on Massive Open Online Courses (MOOC) combined with PBL and Case Study Based Learning (CBL), and used Tencent Conference, WeChat Group, and the first hospital network Bb platform as auxiliary teaching tools. ① MOOC teaching: Students were required to obtain the course content from the teacher before the class began. To improve student participation, videos of case questions and related nursing operations were included in the teaching content of each chapter, and knowledge points and test questions related to case questions were posed to facilitate student understanding and retention. Using a backstage management page, teachers were able to oversee students’ video viewing, learning duration, and test results at any time. They also dynamically mentored students’ participation and learning, and provided targeted feedback and supervision. ② PBL teaching: The teacher prepared a teaching plan incorporating ideological and political material according to the requirements of the syllabus, and presented nursing problems. Students searched the literature and consulted data on the problems, organized the contents, made records, and actively participated in discussions by applying their own analysis and thinking. ③ CBL teaching: Teachers collected typical and special clinical nursing cases, prepared and created complete teaching cases, and organized the students into groups to discuss and learn. ④ Bb teaching platform and WeChat group: Relevant information, case analysis, practical teaching, curriculum-related vocabulary, online testing, syllabus, courseware, a question bank, teaching plan, operation videos recorded by teachers, and other teaching materials were published on the Bb network platform. Course information, teaching progress, syllabus, examination arrangement, etc., were published through a class WeChat group announcement. After class, the training room was opened. After the students had practised in their spare time, they worked with other team members. Each group used mobile phones to record and upload an operation video. The students also carried out operator self-comments, team members’ comments, teachers’ comments, determined the existing problems, and compared videos of the teacher’s demonstration and the nursing students’ operations after class. They then analyzed the cause of the problem and suggested modifications. ⑤ Tencent Conference: Using Tencent Conference, supplementary teaching of the missing chapters in MOOC, in-depth analysis, supplementary explanation of the key chapters, difficult points in the course, and online tutorship were conducted. Comprehensive nursing training, technology operation, hospital training for teachers in the classroom, and real-time interaction with students by way of live display were also conducted.

### 2.3. Evaluation methods

Through case teaching, nursing skills as well as ideological and political education were organically integrated, and the teaching effect was evaluated. The evaluation was performed as follows:

(1) Comprehensive nursing training assessment: This was conducted in the form of an operation examination, where the assessment was divided into three parts. Theory accounted for 10 points. The examination papers for the Comprehensive Nursing Training were drawn from the question bank according to three levels: easy, medium, and difficult. Additionally, part of the papers aimed to evaluate the theoretical knowledge level of nursing students after learning. Skills accounted for 70 points, and students’ operation skill level was evaluated according to the scoring standard of each operation in the teaching material for the Comprehensive Nursing Training. Three teachers participated in the operation assessment to ensure fairness. Humanistic care accounted for 20 points.
(2) The self-study ability of nursing students before and after training was evaluated using the Self-Learning Ability Assessment Scale for Nursing Students compiled by Lin and Jiang (18). The scale is composed of three dimensions with 28 items in total. The three dimensions are self-management ability (including the ability to determine learning needs, time management ability, and learning monitoring ability), information acquisition ability (including the ability to obtain information, expand information channels, and information analysis and processing ability), and learning cooperation ability (including the ability to communicate and to seek help). The Likert 5-level scoring method was adopted, with a scoring range of 28–140 points. In this method, the self-management ability score accounted for 10–50 points, information acquisition ability for 11–55 points, and learning cooperation ability for 7–35 points. The higher the score, the better the autonomous learning ability. The scale has good content validity and structure validity, with a Cronbach’s α coefficient of 0.86.
(3) Assessment using the nurse professional identity rating scale. This scale includes 30 items in the five following dimensions: professional cognitive evaluation, professional social support, professional social skills, professional frustration coping, and professional self-reflection (19). From ‘very inconsistent’ to ‘very consistent’, the scale has a total score of 30–150 points, with a total score of 30–60 points indicating very low professional identity, 61–90 points indicating low professional identity, 91–120 points indicating a medium level of professional identity, and 121–150 points indicating high professional identity. The total Cronbach’s α coefficient for this scale is 0.938 and the split-half reliability is 0.88, indicating its high validity and reliability.
(4) Subjective satisfaction survey of the teaching mode: This survey was a self-designed satisfaction questionnaire, which included six dimensions: course content, teacher teaching, student learning, teacher-student interaction, evaluation, and platform support. The meaning and content of each dimension are defined as follows: Dimension 1: Course content. This refers to the knowledge, skills, concepts, tasks, or activities presented to the students; Dimension 2: Teacher’s teaching. This refers to the teacher’s explanation, demonstration, and guidance of knowledge, skills, concepts, tasks, and other aspects of the teaching process. Dimension 3: Learner’s learning. This refers to the attitude, motivation, engagement, and behavior of the learner during the learning process. Dimension 4: Interaction. This refers to the interactions between teachers and students and among students during the online teaching process. Dimension 5: Assessment. This refers to the assignments, tests, examinations, etc., that students were required to complete to consolidate, test, and evaluate their learning. Dimension 6: Platform support. This refers to the learning environment and support provided by online teaching, including learning resources, retrieval functions, and other services (20). The highest possible score is 100 points. A score below 75 points indicates no satisfaction, 75–89 points indicates satisfaction, and 90 points or above indicates high satisfaction. Satisfaction score = (number of participants with high satisfaction + number of satisfied people)/total number of people × 100%.

### 2.4. Survey method

All questionnaires were completed anonymously. The online questionnaires were distributed and completed using the ‘Questionnaire Star’ online platform. The ‘Questionnaire Star’ survey method not only ensures the integrity of the questionnaire but also enables the participants to fill in the questionnaire in a more natural way, improving the authenticity of the questionnaire results. The survey was sent by professional researchers to the students at the Nursing College of Capital Medical University in Beijing through the ‘Questionnaire Star’ platform. Within 10 days of the comprehensive practical training and assessment of the nursing students, all participants were asked to complete the online questionnaires. Informed consent was obtained at the beginning of the questionnaire. Quality control was carried out by limiting the number of answers and by limiting users with the same internet protocol address to answer only once. A total of 60 questionnaires were issued. All 60 questionnaires were recovered (effective recovery rate 100.00%) and all were valid.

### 2.5. Statistical methods

Epidata 3.0 was used for data entry and SPSS 24.0 was used for data analysis. The Shapiro–Wilk test was used to test the normality of measurement data. All variables were tested for normal distribution. Measurement data in line with a normal distribution were expressed as ( $\bar{x}$ ± s). Independent sample *t*-tests or Pearson chi-square tests were used to compare the demographic characteristics of the intervention and control groups, including age, sex, and nationality. Frequency data (percentage) were analyzed using chi-square tests. Paired sample *t*-tests were used to compare the mean scores on questionnaires. A *p* value <0.05 was considered statistically significant.

## 3. Results

### 3.1. Baseline characteristics of the two student groups

The observation group included 10 males and 20 females, with an average age of 19.87 ± 0.98 years. Of these, 28 were of Han nationality and two of ethnic minorities. The control group consisted of 8 males and 22 females, with an average age of 19.65 ± 0.74 years. Of these, 25 were of Han nationality and 5 were of ethnic minorities. There was no significant difference in the basic data between the two groups (*p >* 0.05) (Table 1).

**Table 1:** General baseline characteristics of the two groups of students.

Variable	Observation group (*n* = 30)	Control group (*n* = 30)	*t*/*X*^2^	*p* value
Gender			0.218	0.640
Male/Female	10/20	8/22		
Average age (‾X ± S, year)	19.87 ± 0.98	19.65 ± 0.74		
Nationality			0.220	0.639
Han nationality	28	27		
Non-Han nationality	2	3		
Residence			0.670	0.795
Town	16	17		
Countryside	14	13		

### 3.2. Comparison of assessment results for comprehensive nursing training between the two groups

Compared with the control group, the students in the observation group had higher scores in venous blood sample collection (84.01 ± 0.87 vs. 92.14 ± 1.23), intradermal injection (91.41 ± 0.82 vs. 96.86 ± 0.27), CPR (87.56 ± 0.31 vs. 93.91 ± 2.79), sputum aspiration (88.11 ± 0.51 vs. 93.75 ± 0.29), and putting on and taking off robes (82.29 ± 0.29 vs. 90.96 ± 0.34), with statistically significant differences (*p* < 0.001), as shown in Table 2.

**Table 2:** Comparison of assessment results of comprehensive nursing training between the two groups of nursing students (X ± S, score).

Group	n	Venous blood sample collection	Intradermal injection	Cardiopulmonary resuscitation (CPR)	Sputum aspiration	Put on and take off isolation clothes
Observation group	30	92.14 ± 1.23	96.86 ± 0.27	93.91 ± 2.79	93.75 ± 0.29	90.96 ± 0.34
Control group	30	84.01 ± 0.87	91.41 ± 0.82	87.56 ± 0.31	88.11 ± 0.51	82.29 ± 0.29
*t*		31.801	33.612	41.301	11.105	12.571
*P* value		<0.001	<0.001	<0.001	<0.001	<0.001

### 3.3. Comparison of scores of independent learning ability between the two groups

Compared with the control group, the students in the observation group had higher scores in self-management ability (33.59 ± 6.34 vs. 37.96 ± 2.73), information acquisition ability (20.14 ± 2.28 vs. 30.68 ± 3.21), learning cooperation ability (27.06 ± 1.20 vs. 31.70 ± 2.19), and total score (82.98 ± 4.72 vs. 93.17 ± 5.01), with statistically significant differences (*p <* 0.001), as shown in Table 3.

**Table 3:** Comparison of scores of independent learning ability between the two groups of nursing students (X ± S, score).

Group	*n*	Self-management ability	Access to information	Learning and cooperation ability	Total score
Observation group	30	37.96 ± 2.73	30.68 ± 3.21	31.70 ± 2.19	93.17 ± 5.01
Control group	30	33.59 ± 6.34	20.14 ± 2.28	27.06 ± 1.20	82.98 ± 4.72
*t*		3.973	4.126	7.130	12.013
*P* value		<0.001	<0.001	<0.001	<0.001

### 3.4. Comparison of occupational identity scores between the two groups

Compared with the control group, the students in the observation group had higher scores in occupational cognitive evaluation (23.59 ± 2.4 vs. 28.16 ± 2.37), occupational social support (22.14 ± 2.82 vs. 26.06 ± 2.31), occupational social competence (21.06 ± 2.10 vs. 27.12 ± 2.27), coping with occupational frustration (22.28 ± 3.72 vs. 26.17 ± 4.01), occupational self-reflection (13.10 ± 0.91 vs. 17.12 ± 1.21), and total scores of occupational identity (102.17 ± 11.98 vs. 124.63 ± 12.17), with statistically significant differences (*p* < 0.001), as shown in Table 4.

**Table 4:** Comparison of scores of occupational identity between the two groups of nursing students (X ± S, score).

Group	*n*	Occupational cognition evaluation	Professional social support	Professional social skills	Professional frustration coping	Professional self-reflection	Total score of professional identity
Observation group	30	28.16 ± 2.37	26.06 ± 2.31	27.12 ± 2.27	26.17 ± 4.01	17.12 ± 1.21	124.63 ± 12.17
Control group	30	23.59 ± 2.43	22.14 ± 2.82	21.06 ± 2.10	22.28 ± 3.72	13.10 ± 0.91	102.17 ± 11.98
*t*		6.715	5.612	7.730	6.510	4.172	23.081
*P* value		<0.001	<0.001	<0.001	<0.001	<0.001	<0.001

### 3.5. Comparison of subjective satisfaction of the two groups with the teaching mode

The subjective satisfaction of nursing students in the observation group was higher than that in the control group (96.67% vs. 90.00%), with a statistically significant difference (*p* < 0.001), as shown in Table 5.

**Table 5:** Comparison of subjective satisfaction of the groups of nursing students with the teaching mode.

Group	*n*	Very satisfied	Satisfied	Dissatisfied	Satisfaction	*χ* ^2^	*P* value
Observation group	30	20 (66.67)	9 (30.00)	1 (3.33)	26 (96.67)	73.329	<0.001
Control group	30	15 (50.00)	12 (40.00)	3 (10.00)	18 (90.00)		

## 4. Discussion

This study examines the benefits of the application of a ‘Internet+’ hybrid teaching mode in a comprehensive nursing training course in the post-epidemic era. The results showed that nursing students in the observation group had significantly higher basic theoretical knowledge and operational skills compared with those in the control group. This study provides references and guidelines for exploring how to combine Internet and teaching reforms to improve the efficiency and effective practice of nursing teaching efforts in the post-epidemic era.

### 4.1. ‘Internet+’ hybrid teaching mode for improving the nursing skills of nursing students

Nursing is a discipline with strong practicality and application. Clinical practice ability refers to the use of professional knowledge and skills to solve practical clinical problems. Clinical practice ability directly affects the quality of nursing as well as patient safety and is also a key indicator of the quality of nursing education and clinical teaching (21). In traditional training classes, students often only have time to complete basic and imitative project operations. To reach the standard proficiency level or to master difficult skills, students need to practise repeatedly after class (22). In the process of practise after class, it is easy for students to forget some details of the operation, but without the timely guidance of teachers, it is impossible to ensure standardization of the operation. The ‘Internet+’ comprehensive nursing training course provided to the observation group made comprehensive use of the school’s Internet Bb and WeChat platforms to carry out mixed teaching exploration and practise. The schoolteachers and clinical front-line teachers jointly filmed a nursing operation video based on the nursing clinical work process in the post-epidemic era, providing students with a reference for pre-class preview, classroom training, and post-class practise. After class, students were able to contact each other in groups and upload videos to WeChat groups using mobile phones, enhancing their enthusiasm for learning.

### 4.2. ‘Internet+’ hybrid teaching mode for improving nursing students’ self-learning ability

In the post-epidemic era, the purpose of mixed teaching of comprehensive nursing training courses is to combine the benefits of online teaching with traditional teaching to improve the teaching quality. This returns the learning initiative to students. With the help of the post-epidemic era and 5G technology, the online and offline hybrid teaching mode presents significant advantages for the teaching of ‘comprehensive nursing training’ (23). A teaching mode that combines PBL, CBL, and online teaching can enhance students’ enthusiasm and initiative and can cultivate students’ logical clinical thinking. Students can communicate and interact with multiple subjects in multiple spaces, can find, analyze, and solve problems through various activities, promoting emotional perception and the internalization of knowledge as well as enhancing critical and innovative thinking (24). This teaching method is expected to take over from the traditional classroom. The research observation group used the online and offline hybrid teaching mode, based on MOOC, combined with PBL and CBL teaching methods, and used Tencent Conference, WeChat Group, and the first hospital network Bb platform as auxiliary teaching tools. The auxiliary tools were used to integrate ideology and politics into the nursing work scene, to identify appropriate ideological and political elements, to deeply analyze students’ psychological characteristics, learning needs, value orientation, and growth laws, and to trigger student knowledge resonance and emotional resonance. Resonance (25) can not only stimulate students’ active participation and change passive learning to active learning but can also improve the quality of classroom teaching and the effectiveness of education (26). The formation of a new teaching mode and teaching framework, where students can guide students to improvement on an ideological level while pursuing personalized values, gives play to the ideological, political, and educational function of the curriculum (27), promoting the comprehensive development of students (28).

### 4.3. ‘Internet+’ hybrid teaching mode for improving nursing students’ professional identity

Professional identity is the psychological basis for individuals to do their job well and achieve organizational goals (29). Individuals with a high sense of professional identity tend to invest more energy in their profession, which is conducive to the stability of professional talent teams. At present, the social status of nursing in China is low, and the public’s understanding of the nursing industry is mostly ‘night shift, busy work, doctor assistant’, etc. Nursing students are generally not exposed to clinical work, and their understanding of the profession comes from the public’s understanding. This view is consistent with the results of this study. The professional identity of nursing students in the observation group was higher than that in the control group. Through the ‘Internet+’ hybrid teaching mode of comprehensive nursing training, teachers in the observation group can transmit information in real-time *via* the network platform, can carry out teaching research on social hot issues, and can use the online and offline hybrid teaching mode to carry out practical teaching activities and break through the space–time constraints of teacher-student interactions to improve students’ learning initiative, broaden students’ vision, and strengthen the effect of practical teaching. When students encounter relevant problems in social practise, they can connect with the instructor in real-time so that problems are solved promptly. Through the network platform, we can provide positive opinions and comments to students, guide students to carry out online speeches and debates according to textbooks and social hotspots, improve the attractiveness of the practical teaching of ideological and political courses, and guide students to establish correct values. Moreover, we can add real anti-epidemic cases to the teaching plan case library and integrate clinical ideological and political elements into the teaching, including the process, protection, and concerns of nursing skills operation in epidemic scenarios. Real cases and teaching pictures/videos let nursing students contact the clinic in advance, let them have a solid and intuitive understanding of clinical work, highlight the professional value of nursing, and help nursing students break through the inherent thinking of the nursing profession and form their own professional cognition. In this study, the nursing students completed the operation independently in groups and recorded a video. By comparing the operation videos recorded by teachers, the nursing students found their own problems, repeatedly discussed with classmates, and answered questions with teachers through the network platform, which helped to improve their sense of achievement and professional identity (30).

### 4.4. Improved student satisfaction with the teaching of comprehensive nursing training courses

The satisfaction of the observation group was significantly higher than that of the control group. The observation group was more satisfied with the teaching form, teaching process, teacher-student interaction, and learning effect than the control group. The ideological and political elements contained in the comprehensive nursing training course allow the cultivation of students’ ideological beliefs, political awareness, and moral and cultural qualities throughout the entire teaching course as well as during effect evaluation. ‘Internet+’ comprehensive nursing practice teaching was employed using the network, which improved the nursing students’ cognition, teaching research, implementation, and response to the ‘Internet+’ teaching mode (31). As a new teaching method, it can effectively improve the enthusiasm and subjective initiative of nursing students, strengthen their curiosity about this teaching mode, and enhance their learning. With the help of the network platform, students can carry out various forms of classroom learning, such as induction, reporting, demonstrations, debates, etc. By recording their own operation videos and reviewing them for self-evaluation, peer evaluation, teacher evaluation, etc., nursing students can check their own gains and shortcomings, teachers can provide targeted guidance and supplementary instructions, strengthening students’ learning of nursing practice. The nursing students appreciated the ‘Internet+’ hybrid teaching model for comprehensive nursing training courses.

This study has some research limitations. First, the sample source was relatively limited and some selection bias might have been present, which makes extrapolation difficult. Second, this study only focused on the effect of ‘Internet+’ teaching on nursing students and has not considered the impact of students’ families on the teaching effect. Finally, research on ‘Internet+’ teaching for nursing management has not yet determined whether it also impacts the learning of other courses, and this requires further exploration in the future.

## 5. Summary

In conclusion, the establishment of the ‘Internet+’ platform has certainly played an intermediary role in nursing teaching in the post-epidemic era. Nursing educators should monitor the teaching quality of nursing students in the post-epidemic era and should combine PBL and CBL teaching methods based on MOOC. Additionally, they should acknowledge the main role of students, mobilize students’ learning initiative, enthusiasm, and creativity, and improve students’ concentration during course learning. Furthermore, they should cultivate students’ clinical thinking ability to maximize the optimization of the teaching and to stimulate the professional identity of nursing students in the post-epidemic era.

## 6. Recommendations

The hybrid teaching mode of ‘Internet+’ comprehensive nursing training can improve the practical operation ability of nursing students and their automatic learning ability and can cultivate their comprehensive ability. Teachers can choose appropriate teaching platforms based on the characteristics of the curriculum in order to improve classroom teaching.

## Data availability statement

The original contributions presented in the study are included in the article/supplementary material, further inquiries can be directed to the corresponding author.

## Ethics statement

The studies involving human participants were reviewed and approved by the ethics committee of Xuanwu Hospital, Capital Medical University. The patients/participants provided their written informed consent to participate in this study.

## Author contributions

Q-LW and L-LL conceived the study. C-RL, Q-SZ, Z-YR, and T-TG participated in its design and coordination. C-YZ, Q-PL, XC, and B-RH helped to draft the manuscript. All authors read and approved the final manuscript.

## Funding

This work was supported by the Capital Medical University Teaching Reform Project: Discussion on “Internet plus” comprehensive nursing training course - mixed teaching mode in the post epidemic era (2022JYY454).

## Conflict of interest

The authors declare that the research was conducted in the absence of any commercial or financial relationships that could be construed as a potential conflict of interest.

## Publisher’s note

All claims expressed in this article are solely those of the authors and do not necessarily represent those of their affiliated organizations, or those of the publisher, the editors and the reviewers. Any product that may be evaluated in this article, or claim that may be made by its manufacturer, is not guaranteed or endorsed by the publisher.
